# Yellow Fever: Origin, Epidemiology, Preventive Strategies and Future Prospects

**DOI:** 10.3390/vaccines10030372

**Published:** 2022-02-27

**Authors:** Elena Gianchecchi, Virginia Cianchi, Alessandro Torelli, Emanuele Montomoli

**Affiliations:** 1 VisMederi Srl, Strada del Petriccio e Belriguardo, 35, 53100 Siena, Italy; virginia.cianchi@vismederi.com (V.C.); emanuele.montomoli@vismederi.com (E.M.); 2 IQVIA Biotech, 1700 Perimeter Park Dr, Morrisville, NC 27560, USA; alessandro.torelli87@gmail.com; 3 Department of Molecular and Developmental Medicine, University of Siena, Via Aldo 3, 53100 Siena, Italy

**Keywords:** yellow fever, infectious diseases, epidemiology, prevention, vaccinations

## Abstract

Yellow fever (YF) virus still represents a major threat in low resource countries in both South America and Africa despite the presence of an effective vaccine. YF outbreaks are not only due to insufficient vaccine coverage for insufficient vaccine supply, but also to the increase in people without history of vaccination living in endemic areas. Globalization, continuous population growth, urbanization associated with inadequate public health infrastructure, and climate changes constitute important promoting factors for the spread of this virus to tropical and subtropical areas in mosquito-infested regions capable of spreading the disease. In the present review, we focus on the origin of the virus and its transmission, representing two debated topics throughout the nineteenth century, going deeply into the history of YF vaccines until the development of the vaccine still used nowadays. Besides surveillance, we highlight the urgent need of routine immunization and vaccination campaigns associated to diverse and innovative mosquito control technologies in endemic areas for YF virus in order to minimize the risk of new YF outbreaks and the global burden of YF in the future.

## 1. Introduction

Yellow fever (YF) is a mosquito-borne viral illness caused by an arbovirus of the family *Flaviviridae*, genus *Flavivirus*, encompassing positive-single-stranded RNA viruses. The virus was isolated for the first time in 1927 in a male patient [1]. Transmission is primarily by mosquitoes [2]. After an incubation period of 3–6 days, YF infection can cause the onset of different clinical features, ranging from a self-limited or mild febrile illness with flu-like symptoms in most of the cases to severe hemorrhage and liver disease. The analysis of data on asymptomatic infections, mild disease, severe disease (fever with jaundice or hemorrhagic symptoms), and fatalities collected in 11 studies involving Africa and South America during the period 1969–2011 was used by the group of Johansson [3] to estimate the probability of each infection outcome. In more detail, in cases of YF virus infections, the probability of being asymptomatic was 55%, whereas the probabilities of developing mild and severe diseases were 33% and 12%, respectively. The probability of death for people experiencing severe disease was 47%. Symptoms include fever, headache, jaundice, muscle pain, nausea, vomiting, and fatigue. Such variety in the clinical spectrum makes YF diagnosis difficult. In those patients presenting a severe infection, hemorrhagic fever can develop leading to the death of the infected subjects. The Case fatality rate has been estimated as 20–50% in patients with severe symptoms [4], accounting for ∼78,000 deaths every year, although misdiagnosis and under-reporting might be responsible for underestimation of the mortality rate [5].

## 2. The Origin and Transmission of YF

### 2.1. The Origin of YF

The origin and transmission of YF represented two debated topics throughout the nineteenth century.

At the beginning, it was sustained that YF originated from the Americas, wherein it was discovered at the end of the fifteenth century by the first Spanish conquerors. In fact, the first identification of YF in America was due to circumstantial reasons: for economic reasons and due to the presence of settlements, the New World drew more attention with respect to Africa. From the middle of the seventeenth century, many epidemics were registered in America as well as in the West Indies. Although the first formally identified YF epidemic in history dates to 1647, the year in which an YF epidemic occurred in Guadeloupe, the reference of putative or plausible cases before then caused people to identify YF presence in the New World before the Spanish conquerors arrived [6].

Epidemiological and genetic studies sustain the hypothesis that the YF virus originated in Africa [7] and would be introduced in the 16th century by the trading of slaves from endemic African countries into countries of the Western region of America, causing outbreaks there between the 17th and 18th centuries [8].

Two points of evidence support the theory that the crews of Columbus would have introduced YF virus for the first time in America between 1492 and 1495 from the Canary Islands, the region where the ships of Columbus made their last resupplying visits before reaching the New World. The first observation is that the disease was noted a few months after the battle launched by Christopher Columbus against the Amerindians in 1495 in Hispaniola (today known as Dominican Republic), and the second point of evidence is that the infection was referred to with different names on the basis of the very recognizable symptoms by European navigators navigating along the African coast and the Canary Islands as far back as 1494, regardless of the discovery of the Americas. In addition, the analysis of the length and the conditions of the journey, which likely did not allow patients to survive the journey to the Americas [9], sustain the hypothesis of a role played by *Aedes aegypti*’s eggs in introducing YF in the New World through transovarian virus transmission being able to stay alive for many months when desiccated [10].

Molecular investigations have highlighted a more marked genetic heterogeneity of YF in Africa supporting here its origin [6]. At the turn of the 19th century, YF was a known and feared pestilence in the western hemisphere and coastal regions of West Africa, whose etiology and mode of transmission were unclear. Known as “yellow jack” because of the yellow quarantine flag on ships, the disease long terrified people and disrupted trade. Although little was known about the disease, it occurred in an epidemic and endemic form and was associated with ports. In fact, new outbreaks were often accompanied by the arrival of ships [11].

Comparing the number of epidemics in the two continents occurred between the 17th to the 19th century, it can be observed that the Americas were hit by a higher number due to a set of ecological, socio-economic, and demographic conditions [6].

The highly populated cities of the eastern coast of the United States (US) constituted a favorable condition for the spread of YF virus imported by ships from the Caribbean, with repeated epidemics in the US occurring in cities such as New York City, Philadelphia, Baltimore, and New Orleans in the 18th and 19th centuries, causing the death of hundreds of thousands of people in America [12]. Conversely, in Africa, the reduced population density did not promote YF spread [6]. After 1822, YF cases were limited to the South US.

YF outbreaks had important consequences not only for public health but also for geopolitics and the economy. More in detail, as occurred in the Spanish–American War of 1898, YF was responsible for a higher number of deaths in the volunteer troops respect the war itself [13], whereas between 1904 and 1914, YF caused the delay in completing Panama Canal construction due to the thousands of deaths [14].

### 2.2. The Transmission of YF

Concerning YF transmission, from the seventeenth century until the end of the nineteenth century, it was argued that it could occur by water and/or human contacts, sustaining the idea that the germ penetrated the body though the respiratory system. Following the epidemics that occurred in Philadelphia in 1793, Cadiz in 1800, and Barcelona in 1821–1822, wherein the absence of direct contact between the patients could not have had a role in their spread, the hypothesis regarding the modality of disease transmission progressively changed at the end of the eighteenth century, sustaining the idea that direct contamination between people could not be responsible for YF transmission. The first experiments that shed some light in YF transmission were those conducted by Walter Reed and his colleagues [15]. Reed’s research built on what Carlos Finlay, a Cuban physician and scientist, had discovered in 1881. Finlay advanced the research into the mode of transmission, as he suggested that *Culex cubensis* (now known as *Aedes aegypti*) might be the mosquito responsible for spreading the disease [16]. At the end of the 19th century, the US invaded Cuba during its war with Spain. For every soldier who died in battle, thirteen fell ill and died of YF [16]. For this reason, Walter Reed and his colleagues were sent by Surgeon General George Sternberg to Cuba to investigate the causes of the disease. Reed’s work proved what had been argued years earlier by Finlay and that the disease was caused by a filterable agent found in the blood of infected patients [15]. In addition, Reed’s work led General William Godas to conduct campaigns against the urban mosquito vector, eliminating that disease in 1902 [17], and the same process was carried out in Panama 4 years later.

## 3. YF Epidemiology and Transmission Cycles

Mosquitoes able to transmit YF virus belong to the *Aedes* spp. in Africa and *Haemagogus* spp. or *Sabethes* spp. in South America. YF virus is currently endemic in 34 countries in Africa and 13 in South America [4]. YF virus can be classified into two principal clades. In more detail, the first clade encompasses four genotypes, two in West Africa and two in South America, and it has been supposed that such divergence between African and South American genotypes may have arisen about 470 years ago [16], whereas the second clade includes three genotypes identified in Central/East Africa [18]. The oldest strain is represented by the East African, which is likely originated from an ancestral flavivirus roughly 3500 years ago; afterwards, West African strains diverged from East African ones approximately three centuries before YF introduction into the Americas. A stricter similarity of American strains to West African strains has been observed compared to the similarity between the latter and the East African strains (rev. in [6]).

The virus is maintained in nature by transmission between non-human primates (NHP), horizontal transmission via blood-feeding mosquitoes, and transovarial transmission in competent vectors. Since NHPs represent one of the reservoirs, the YF virus cannot be eradicated. In addition, infected humans can also contribute to the transmission of the virus infecting mosquitoes during periods of viremia and spreading the virus. It has been estimated that YF virus causes 200,000 cases of disease and 30,000 deaths every year, 90% of them in Africa [19].

The identification of vectors in different habitats has led to the establishment of three distinct transmission cycles: wild, semi-domestic, and domestic.

In more detail, in the case of sylvatic (or jungle) cycle NHP (monkeys) living in tropical rainforests, this represents the principal reservoir of the YF virus, which is transmitted to other monkeys when bitten by wild infected mosquitoes. Humans working or travelling in the forest develop YF when bitten by infected mosquitoes. This type of transmission accounts for most of the cases in South America since 1942 and historically was limited to the Amazonian regions, although it can possibly cause wide outbreaks, as observed in Brazil [20,21].

The semi-domestic (or intermediate) cycle, representing the principal type of transmission and the principal cause of outbreaks in the African savannah, involves humans working or living in jungle border territories bitten by semi-domestic mosquitoes living both in the wild and around households. The virus can be transmitted not only between monkeys but also between humans.

A domestic (or urban) YF cycle is less common, and it occurs when the YF virus is introduced into highly populated areas with elevated mosquito density by infected people that, after having contracted the virus in the jungle or through the intermediate cycle, come back into the urban area. Here, the virus can be transmitted from person to person by competent urban mosquitoes [22] leading to the onset of uncontrolled outbreaks with devastating consequences [23].

**Figure 1:**
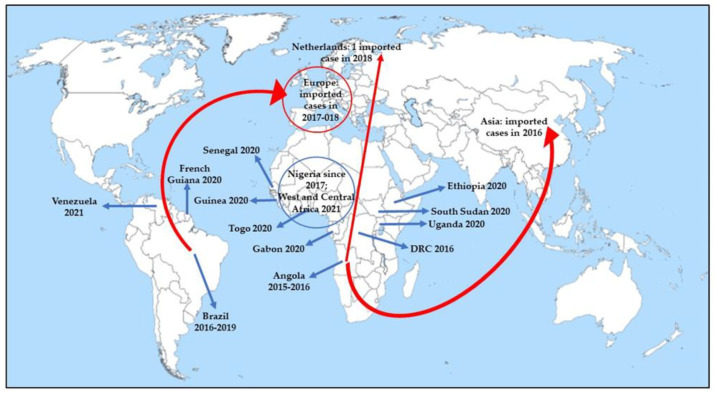
Recent wide autochthonous outbreaks (indicated by blue arrows) and imported YF cases (indicated by red arrows) [24].

Epidemics are currently registered more frequently and important in Africa than in the Americas [6]. Recently, YF outbreaks have involved Uganda (2020), South Sudan (2020), Ethiopia (2020), Guinea (2020), Gabon (2020), Senegal (2020), Togo (2020), West and Central Africa (Cameroon, Chad, Central African Republic (CAR), Côte d’Ivoire, Democratic Republic of the Congo (DRC), Ghana, Niger, Nigeria, and Republic of Congo in 2021), French Guiana (2020), and Venezuela (2021) (Figure 1) [24].

Although the domestic cycle plays a limited role in YF transmission, especially in South America, urban outbreaks have been registered in Angola and DRC [25]. If massive vaccination programs during the 1940–1950s and the 2000s significantly reduced YF virus outbreaks [4], the reduction in vaccination coverage between the 1960s and the mid-2000s led to a rise in YF outbreaks in South America and Africa. YF outbreaks occurred in 2016–2018 in non-endemic areas and in endemic areas historically characterized by reduced YF virus activity, all distinguished by low vaccination coverage, suggest that the YF virus represents a major threat to public health [26,27]. Moreover, the lower routine vaccination coverage in 2020 without catch-up vaccination for YF, as well as for other infectious diseases, due to the ongoing pandemic of Severe Acute Respiratory Syndrome Coronavirus 2 (SARS-CoV-2), could be responsible for an increased number of cases [28]. The creation of the Eliminate Yellow Fever Epidemics (EYE) strategy by the World Health Organization (WHO) aims to eliminate urban YF outbreaks by 2026 [29].

**Figure 2:**
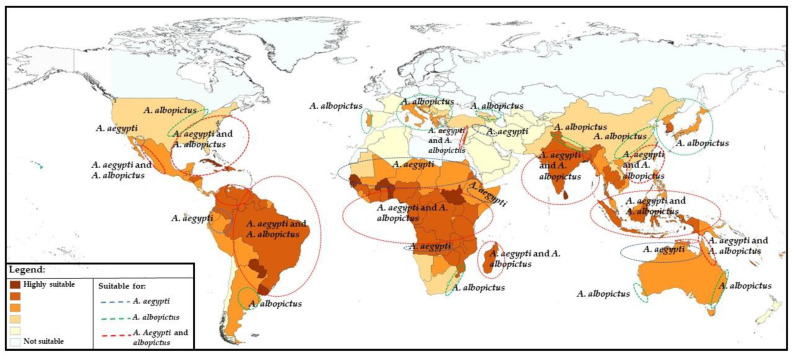
Country suitability for *Aedes aegypti* and/or *A. albopictus* [30].

Although *Aedes aegypti* is also present in the tropical and subtropical regions of Australia, Asia, and the Pacific (Figure 2) [30], neither cases nor outbreaks caused by YF virus have occurred in these regions so far, leading to several assumptions. Among these speculations are the absence in recent centuries of slave trades from endemic countries to Asia [31], the presence of less competent vectors of YF in Asia [32] not allowing YF virus transmission, the presence of other related flaviviruses that might provide cross-protection immunity towards YF, and the lack of or insufficient YF viral load introduced by immigrants from epidemic regions (rev. in [31]).

## 4. Prospects for Changing Epidemiology in Future

As occurred for the outbreaks caused by other infectious diseases whose spread was limited in the past by human actions, such as vaccination, nowadays globalization, the continuous population growth, urbanization associated with inadequate public health infrastructures and climate changes constitute important promoting factors for the spread of not only new infectious agents but also well-known pathogens, including YF virus [33]. In more detail, if in the 1950s and 1960s, urban epidemics of YF were effectively limited by mosquito control, human vaccination, and strict requirement for travelers to and from endemic areas, the reduction in vaccination programs in endemic countries associated with a gradual relaxation of restrictive measures for travelers has played a significant role in promoting the epidemic transmission in some African and American countries [34].

Even though, so far, no autochthonous transmission of YF has been registered in Asia or in Oceania, the population is susceptible to the YF virus, as observed by Asian infected subjects living in African or South American endemic regions [6].

The first cases of YF imported from an outbreak occurred in Angola into Asia were reported in 2016 (Figure 1) [24,35]. As observed for other infectious diseases, such as chikungunya and Zika virus, whose increased incidence was promoted by human intervention, including climate change, urbanization, and unsustainable vector control, they could play a critical role in promoting the diffusion of the YF competent vector, widening its habitat [36]. A risk analysis assessment conducted by the group of Daniels regarding the introduction of YF via air travel into Asia in 2016 [37] reported that although there is an increase in air travel, and 25 Asian cities were identified as at risk of receiving at least one YF viraemic traveler during 2016, the risk of YF local transmission in Asia during 2016 due to introduction from endemic countries was limited. This finding was in accordance with the absence of autochthonous transmission in the continent so far, allowing us to hypothesize the role played by several factors, such as biological, environmental, and societal factors, in preventing such transmission [38]. It is very likely that: (1) the fact that Asian *Aedes aegypti* is relatively incompetent in transmitting YF, (2) weak adaptation of the vector to humans, (3) competition between vectors, (4) competition between *Flaviviridae* in the vector as observed by in vitro studies, wherein dengue virus interferes with the YF virus infection [39] and replication within the mosquito cells [40], (5) cross-reactivity with other *Flaviviridae*, and (6) the development of some forms of cross-immunity between *Flaviviridae*, and in particular, between dengue and YF, could have played a role in elucidating YF loss in Asia [6].

As an increase of 5% per year in travelling to Asian countries has been estimated to occur, YF introduction through viremic travelers and autochthonous transmission into new areas can rise, representing a threat for public health [37].

However, in the last 20 years, YF virus epidemiology has changed, as demonstrated by the recent wide outbreaks that occurred both in South America (Brazil in 2016–2019) and Africa (Angola in 2015–2016 and DRC in 2016) based on urban vector transmission in the last years [41,42] and previously identified as low-risk regions. In Brazil, the expansion of regions wherein YF was endemic, whose reasons remain to be elucidated, had led to a reassessment of YF endemicity in this country with the inclusion of five new regions in 2000 and the entire country since 2018. Although YF seasonality has been identified [41], limited information regarding the role of environmental and climatic factors associated to seasonality in South America are currently available.

The study of habitat suitability for *Aedes aegypti* and *Aedes albopictus* has highlighted the identification of highly suitable areas for both in the southern USA, Caribbean, South America, Sub-Saharan Africa, Indian subcontinent, Southeast Asia, and some Pacific countries. In addition, patchy foci of suitable areas were detected in countries of Southern Europe and North Africa along the Mediterranean coast, as well as in Israel and in the territories along the Euphrates and Tigris rivers and the coastal areas of northern Australia (Figure 2). On the contrary, the tropical and sub-tropical parts of the world represent the most suitable habitat for *Aedes aegypti*; suitable areas for *Aedes albopictus* are more extended, also including the temperate part of the world such as Southern Europe and central USA [30].

The recent study conducted by Hamlet et al. [43] evaluated the contribution of vegetation, land cover, climate, and host population in predicting the numbers of months wherein YF cases were reported, considering the period 2003–2016. The research supports that vegetation type and heterogeneity (representing maybe a marker for habitat fragmentation) and land cover have a role in the trends in YF transmission. Even though a putative link between vegetation type and population exists, with anthropogenic activities leading to long-term consequences for the vegetation type, the study did not allow us to find a correlation. As sustained for other zoonotic disease transmission, but so far not statistically recognized in YF emergence [44], fragmentation and vegetation heterogeneity could be implicated in YF epidemiology. In more detail, fragmentation could influence sylvatic hosts in several ways, which include the promotion of their contacts to humans via modified behaviors [45,46] or enhancing infection susceptibility due to a stress-weakened immune system [47]. In addition, vegetation heterogeneity can influence vector dynamics, promoting spillover through higher human–sylvatic cycle contact or promoting the presence of more anthropophilic vector species in fragmented habitats [48].

Sadeghieh [49] assessed if future climate changes could impact YF virus outbreaks in Brazil by modifying mosquito ecology. The study used simulations from regional climate models considering three time periods: 2011–2040 (short-term), 2041–2070 (mid-term), and 2071–2100 (long-term), suggesting that climate change has an effect on mosquito-borne diseases. More specifically, YF outbreaks could diminish in intensity as temperatures increase in Brazil, not allowing *Haemagogus* mosquito survival, although temperature is not the only factor exerting an impact on disease transmission. Concerning the contribution of climate changes on YF, this relationship is probably complex, and it is plausible that climate modification could have a role in the worldwide distribution of the vector, with a higher risk in those regions wherein climate modifications are more likely to occur. At the same time, as predicted for Brazil, the risk of outbreaks could diminish in endemic regions because of altered seasonal temperature [50].

A potential explanation regarding the recent outbreak that occurred in Brazil has been provided by Haslwanter [51]. In greater detail, in his study, he reported a reduced antiviral potency of the polyclonal antibody response in vaccines against an emergent Brazilian strain due to genotype-specific features that are unique to and characterize most South American YF virus strains. This observation supports the re-evaluation of current approaches to YF virus immunological surveillance in South America, suggesting the necessity of updating vaccines, since the current ones are based on a live-attenuated YF-17D virus derived from the virulent African isolate.

The identification of invasive *Aedes* spp. in North America has also increased concerns in that area. In fact, in addition to the rising amount of arboviral (Chikungunya and Zika) imported cases, locally acquired arbovirus infections have been reported in the US [52].

More specifically, predictive climate models based on rain fall, temperature, and winter survival capacity support the establishment of this vector in southern Canada by 2040 [53]. Ongoing monitoring with the aim to identify an elevated number of different species of mosquitoes would be fundamental for public health authorities [54].

Concerning Europe, whereas *Aedes albopictus* has been identified in Southern and continental France since 2004 [55], the majority of *Aedes* spp.-related arboviral are imported, although certain areas have favorable conditions for vector survival and reproduction, suggesting that YF is a potential emerging disease of considerable importance. Imported cases of YF have been reported in European countries (France, Netherlands, Germany, Romania and Switzerland (one imported case each) in 2017–2018 (Figure 1) [52].

## 5. YF Vaccine

### 5.1. The History of the YF Vaccine

After the First World War, The Rockefeller Foundation established a YF Commission with the aim of eradicating the disease through the elimination of *Aedes aegypti* [17]. Although these initial activities had a positive impact against urban YF (*Aedes aegypti*-borne), the goal of eradicating the disease fell through with the discovery that YF was a zoonosis, kept by sylvatic mosquito species and NHPs in the Amazon jungle [56]. In 1927, Adrian Stokes isolated the virus from the blood of a sick man in Ghana known as Asibi. Three years later, Max Theiler was able to identify mice as animal models, as they were susceptible to intracerebral virus inoculation. Theiler and his colleagues passaged the Asibi virus more than 200 times in cell cultures. The test of this subculture, called 17D, showed that the virus had become attenuated, but it could still elicit a protective immune response in monkeys and humans [17].

The 17D vaccine received licensing approval in 1938, with more than 850 million doses distributed since, allowing Theiler to receive the Nobel Prize in Physiology or Medicine for his discovery concerning YF and how to combat it in 1951.

Some months later in 1927, a different strain isolated at the Institute Pasteur in Dakar allowed the development of a second attenuated vaccine. This time, the virus was attenuated by a series of passages in mouse brain, leading to the development of the French neurotropic vaccine (FNV). This vaccine was extensively used in 1960 in francophone Africa, resulting in the disappearance of the disease [57]. These findings formed the basis for further investigations that led to a broader understanding of the ecology, epidemiology, etiology, and prevention of YF. Since the 1940s, mass campaigns with the 17D vaccine have been conducted in South America, and vaccination with FNV became mandatory in French-speaking Africa [57]. In 1950 and 1960, fears were expressed about the high rate of post-vaccination encephalitis in children after FNV, and that vaccine was abandoned in 1982 [58]. Instead, adverse neurological events with the 17D vaccine appeared to be sporadic and affected children younger than 9 months. Since 1988, the WHO and the Pan American Health Organization have promoted the use of YF vaccine in routine child immunization programs [59].

### 5.2. YF Vaccine

Recent decades have witnessed an unprecedented re-emergence of YF virus both in urbanized areas where vaccination coverage is low [60,61] and in high-risk areas where vaccination represents a potential method of prevention for outbreaks [60,62]. These situations highlight the urgent need to improve health surveillance of the disease and expand knowledge about vaccine protection, given the historical era in which international travel represents a daily occurrence.

The YF virus, as mentioned above, was isolated in 1927, and from this time, efforts to produce a vaccine began. The first results, which were not satisfactory, were aimed at the development of an inactivated vaccine, after which the focus was shifted to the development of a vaccine based on live viral products [63]. Currently, the main type of YF vaccine is based on the live-attenuated 17D virus. This vaccine was formulated after numerous passages of the wild-type Asibi strain in embryonated chicken eggs [64]. To date, the WHO has prequalified and stockpiled for use in vaccination programs only four vaccines: the 17D subculture is the seed strain for all the modern YF vaccine, whereas three sub-strains derived from the first 17D vaccine (the 17DD (passage 195), 17D-204 (passage 204), and the 17D-213, derived from 17D-204) [64,65,66]. Small differences in genomic sequences distinguish the different sub-strains; in particular, they vary in glycosylation sites on the envelope protein, although no differences in immunogenicity were noted [67]. The original virus passage 176 no longer exists, and therefore comparisons between strains are only carried out on the aforementioned three sub-strains [68].

Proof of YF vaccination is necessary to travel to some countries according to the International Health Regulations (IHR). For those traveling to endemic areas, vaccination is recommended to protect the traveler’s health [69]. One risk is characterized by unvaccinated travelers who may import the infection into other countries. Concern about the spread of YF in susceptible populations increased when people infected in Angola traveled to/back to countries such as DRC, Mauritania, Kenya, and China [65]. Because the YF vaccine is live attenuated, it is contraindicated for immunocompromised individuals (e.g., HIV-infected individuals or subjects taking immunomodulating medications) [69]. Therefore, in such cases, vaccination takes into account multiple factors, including the traveler’s age, destination, medical history, and immune status [69,70,71,72].

### 5.3. YF Vaccine Efficacy

Individuals vaccinated against YF show high levels of protection with a seroconversion rate greater than 95% in both adults and children [73]. However, it has been seen that children less than two years of age may show a lower level of seroconversion after a single dose of vaccine [74]. The protection conferred by the vaccine has been amply demonstrated over time. In more detail, after vaccination campaigns in South America, a rapid decrease in cases occurred [75,76,77]. In areas where the disease is endemic, populations are protected against infection by the virus due to herd immunity, which means that the high level of protection is a direct consequence of the high percentage of immunized people within this population [78].

A booster dose, initially every 9 months, was enforced in 1959 by the International Sanitary Regulations, the precursor to the IHR [79,80]. The booster period was then changed in 1965 to every 10 years, based on data showing the presence of neutralizing antibodies for at least 10 years after vaccination [81,82]. As of 2011, the WHO Strategic Advisory Group of Experts (SAGE) has concluded that a single dose is sufficient to support sustained protection against YF, without the need for a booster dose. Immunocompromised and immunosuppressed subjects represent the exception for which the booster is necessary [76]. Once SAGE redefined the guidelines stipulated years earlier by IHR, the United States Advisory Group (ACIP) also wanted to determine whether a booster needed to be implemented. Therefore, based on available data, ACIP voted that a single dose confers protection and is appropriate for most travelers [69]. However, as a preventive measure, a dose may be given to those who, having received their first dose 10 years before, travel to high-risk settings or spend prolonged periods in endemic areas. One of the pivotal points underlying a decision whether to administer a vaccine booster is the protective immunity conferred by the vaccine. The protective correlate that exists for this vaccine comes from a study conducted in NHP following YF vaccination and then tested with the wildtype variant of the vaccine [76,83]. Log10 neutralization index (LNI) ≥ 0.7 was considered to be a cut-off for protection. To date, the plaque reduction neutralization test (PRNT) is also used to establish quantitative titer of virus-specific antibodies. In addition, most clinical trials use a PRNT50 assay with a titer of 1:5 as a protective correlate [84,85]. The decision that the vaccine may confer lifetime protection is based on the rarity of failures in vaccinated recipients. In fact, 90% of vaccinated subjects develop neutralizing antibodies within 10 days of administration, and 99% develop neutralizing antibodies within 30 days. The presence of neutralizing antibodies to YF is closely correlated with protection. This concept has raised concerns about whether a single dose of vaccine can protect people with diminished neutralizing antibodies and those traveling to high-risk areas [86,87,88,89,90]. Some doubts were raised by those vaccinated subjects who developed a lower antibody response or showed shorter than average antibody duration [69,86,87,91]. As demonstrated by Lindsey et al., the level of protective neutralizing antibodies after a dose of YF vaccine in 146/150 individuals vaccinated within 10 years was 94% [69,86,87,88]. Further studies have shown that in Brazilian children, the rate of seroconversion was lower when the YF vaccine was received concurrently with other vaccines, such as measles, mumps, and rubella. This condition is possibly related to an interaction given by the co-administration of these two vaccines, both of which are live attenuated [92,93]. Such observations were confirmed by Goujon et al., who found that in a group of 131 children, 4 of them vaccinated with the two aforementioned live attenuated vaccines did not develop a protective antibody titer against YF [93]. Other concerns also arose from the rates of seropositivity in some children, which showed a decrease a few years after YF vaccination. This decrease in seropositivity rates was detected in children living in two African countries, Mali and Ghana. In the first case, in fact, it was seen that the seropositivity rate decreased from 96.7% 28 days after the administration of the vaccine to 50.4% about 5 years later. The same scenario occurred in Ghana, where in some children, the seropositivity rate decreased from 72.7% 28 days after vaccination to 27.8% about 3 years later [94]. Therefore, lower seroprotection and an attenuated immune response have led to more stringent recommendations for both persons vaccinated during childhood and those with compromised immunity (e.g., HIV-infected persons) [69]. For this reason, the ACIP suggests a booster dose after 10 years for some specific categories, including individuals who have received YF vaccination prior to a hematopoietic cell transplant, people who handle YF virus on a daily basis, and those who travel to high-risk areas [69].

### 5.4. Common Symptoms and Adverse Effects to Yellow Fever Vaccine

Mild viremia usually occurs in individuals who have received the first dose of vaccine. It occurs 3–7 days after immunization and lasts about 1–3 days. Increased levels of the cytokines Tumor Necrosis Factor alpha (TNF-α) and Interferon gamma (IFN-γ) and markers of T-cell activation have been detected and may represent the mediators underlying the common effects occurring in vaccinated subjects. Monath et al. also observed that viremia does not occur following subsequent doses of the YF vaccine, and possible side effects appear milder. With the development of neutralizing antibodies, there is also a resolution of viremia [63].

Adverse effects following vaccination are generally mild to moderate and appear 5–10 days after immunization. Among the most common manifestations are myalgia, headache, and fever [95,96]. Cases of severe multiorgan failure after vaccine occurred in the late 20th century. Since that time, the medical community has sought to investigate the causes of adverse events and risk factors for severe vaccine effects. Three types of severe adverse events have been identified: hypersensitivity reactions, neurotropic disease, and viscerotropic disease. Since the YF vaccine is prepared in embryonated eggs, people who are allergic to eggs should not be vaccinated. Gelatin, another component of the vaccine, may also have an impact on triggering an allergic reaction. Systemic allergic reactions, such as anaphylaxis and urticaria, represent a rare event, approximately one case per 58,000 to 131,000 individuals [97]. The vaccine-associated neurotropic disease onset varies from 4–25 days and occurs with fever, headache, and focal neurologic findings, and it was detected from 1945 to 2002 in 23 of more than 200 million vaccinated individuals worldwide [98]. An incidence of one case per 150,000 to 250,000 doses administered was reported within the Vaccine Information Statement, written by the US Centers for Disease Control and Prevention [99]. However, most individuals who become ill recover without complications. The vaccine-associated viscerotropic disease is a syndrome characterized by severity that can be moderate to severe, even leading to death. Symptoms begin 2–5 days after vaccination, and the most common symptoms include fever, elevated hepatocellular enzyme levels, respiratory failure, blood dyscrasia, and sometimes, renal failure. Viscerotropic disease is manifested by an immune response characterized by excessive viral replication and in which the antibody response is elevated [63,100]. A report released by the Vaccine Adverse Event Reporting System in the US has highlighted as a category at risk people of advanced age, although some cases have been detected even among younger people [101]. Pathologies afferent to the thymus are to be considered another possible risk factor for the development of YF vaccine-associated viscerotropic disease. In fact, Eidex BR highlighted in a research study how 15% of a cohort of 26 subjects who developed a viscerotropic disease after vaccination had a thymus pathology, such as myasthenia gravis or thymoma [102]. Within the Vaccine Information Statement, it was reported that the incidence of viscerotropic disease is one case per 200,000–300,000 doses of vaccine administered, while in subjects over 60 years of age, the incidence is higher (one case per 40,000–50,000 doses) [99].

### 5.5. The Issue of YF Vaccine Supply: The Use of Fractioning Doses

Currently, the global stockpile is insufficient to provide full-dose vaccination to people threatened by epidemics. As a result of outbreaks in Angola and DRC that occurred in 2016, the WHO proposed a strategy to Eliminate EYE with the goal of globally eliminating yellow fever outbreaks by 2026. This strategy involves protecting at-risk populations in endemic areas, preventing the international spread of the disease through travel, and containing outbreaks [103]. Since there is no treatment for the disease and no vector control can be applied, added to the fact that the reservoir is not only humans, these factors represent a limitation in eliminating disease transmission. Therefore, to date, the most effective measure to contain YF in populations at risk is vaccination. Mass vaccination programs have, over time, made it possible to deal with outbreaks. However, due to a decline in population immunity, outbreaks have emerged again. In 2001, 6 million doses of YF vaccine were used to respond to the emerging outbreaks. However, the 2015 outbreak in Angola led to a depletion of the emergency vaccine stockpile. The WHO recommended the use of fractionated doses of the 17DD vaccine (containing one fifth (0.1 mL) of the standard dose) to increase the number of available doses by five times in the neighboring country, DRC, where vaccine coverage was low [104]. Each fractioned dose should, in fact, contain at least 1000 IU. Recommendations suggested by the WHO regarding dose sparing are based on two clinical studies, one carried out in The Netherlands and one in Brazil. The Dutch study was a randomized, controlled trial that showed that intradermal administration of a fractionated dose with 0.1 mL was not inferior to subcutaneous administration with 0.5 mL (standard dose) [105]. The initial hypothesis was that the intradermally administered vaccine might be more immunogenic compared with the subcutaneous one because of the direct targeting of antigen-presenting cells in the papillary dermis. However, despite the lower dose of vaccine, a high viremia comparable to those who were given a standard dose was detected in the participants. This may be justified by the fact that, regardless of the route of administration, live vaccines are able to spread rapidly through the body to reach their target cells [106]. The Brazilian clinical trial, on which WHO based its fractional-dose guidelines, is a randomized, controlled trial featuring a design with decreasing doses administered subcutaneously of the YF vaccine. The dose considered as a reference was 27.476 IU with a decrease to 31 IU per vaccine dose [107]. Of those in the lowest dose groups who showed seroconversion in the first month after fractionated vaccination, 98% displayed neutralizing antibody titers after 10 months. However, a limitation for both studies is that the population cohort on which the trials were performed is the traveler category consisting of healthy young adults and not people living in areas endemic for the disease [108].

In June 2017, the WHO declared its official strategy regarding vaccine dose fractionation. The agency recommends the use of fractionated doses during outbreaks only if there is a shortage of vaccine to respond to the emergency. However, for pregnant women and children younger than 2 years of age, a full dose is recommended because of limited data on the safety and immunogenicity of the vaccine. Data from the observational study conducted in Kinshasa by Ahuka-Mundeke with fractionated doses (except for women and children) showed that 98% of them showed seroconversion 1 month after vaccine administration [109]. The promising results derived from the use of fractionated doses has created enormous expectations for the implementation of vaccination for completion before 2026 of the EYE program [110]. Difficulties in facing the emergency of limited YF vaccine stockpiles have led to the design of alternative vaccines. One promising candidate is a plant-produced subunit vaccine derived from a protein in the virus envelope [111]. Although one study demonstrated partial protection in mice, this novel vaccine showed lower efficacy when compared with the live attenuated vaccine [111]. Vaccinating monkeys against YF is another strategy that has been proposed [112], in addition to vector control [113].

## 6. Conclusions

Although YF represents a vaccine-preventable disease, and the vaccine is able to confer effective and long-lasting protection, YF continues to represent a principal public health issue, as demonstrated by the large outbreaks that occurred in the last 30 years, which also spread even recently in new areas, and for this reason, it is considered a re-emerging disease [42]. These outbreaks are not only due to insufficient vaccine coverage for insufficient vaccine supply, but also to the increase in people without history of vaccination living in endemic areas [114]. Moreover, increased travel in a highly globalized civilization creates a high risk of YF spreading to tropical and subtropical areas in mosquito-infested regions capable of spreading the disease. However, in the last few years, vaccine supplies seem to be limited, and therefore much energy have been directed towards finding innovative strategies to compensate for the emergency. In fact, although the animal reservoir of the disease cannot be eliminated to date, the means to eliminate YF infections in humans seem promising.

In light of recent data on YF virus spread, besides surveillance, which is characterized by difficulties due to YF’s broad clinical spectrum and cross-reactivity with other flaviviruses and needs to be increased in resource-limited areas, routine immunization and vaccination campaigns associated with diverse and innovative mosquito control technologies play a fundamental role in endemic areas for YF virus in order to minimize the risk of new YF outbreaks and the global burden of YF in the future. Future long-term previsions regarding the burden of the disease are necessary for correct immunization campaigns.

## Data Availability

Not applicable.
